# 4-Hydrazino-2-(methyl­sulfan­yl)­pyrimidine

**DOI:** 10.1107/S1600536809003286

**Published:** 2009-01-31

**Authors:** Hoong-Kun Fun, Ibrahim Abdul Razak, Adithya Adhikari, Balakrishna Kalluraya

**Affiliations:** a X-ray Crystallography Unit, School of Physics, Universiti Sains Malaysia, 11800 USM, Penang, Malaysia; b Department of Studies in Chemistry, Mangalore University, Mangalagangothri 574 199, Karnataka, India

## Abstract

In the crystal of the title compound, C_5_H_8_N_4_, centrosymmetric dimers are linked by pairs of N—H⋯N hydrogen bonds. Further N—H⋯N links result in a two-dimensional array whereby wave-like supra­molecular chains are inter­connected by *R*_2_^2^(8) ring motifs.

## Related literature

For general background, see: Ghorab *et al.* (2004 ▶); Anderson *et al.* (1990 ▶); Géza *et al.* (2001 ▶); Gante (1989 ▶); Powers *et al.* (1998 ▶); Vidrio *et al.* (2003 ▶). For details of hydrogen-bond motifs, see: Bernstein *et al.* (1995 ▶).
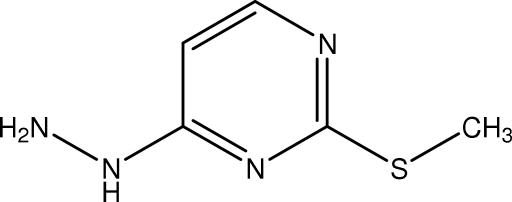


## Experimental

### 

#### Crystal data

C_5_H_8_N_4_S
*M* *_r_* = 156.21
Orthorhombic, 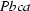

*a* = 12.7906 (2) Å
*b* = 7.7731 (1) Å
*c* = 14.4354 (3) Å
*V* = 1435.21 (4) Å^3^
*Z* = 8
Mo *K*α radiation
μ = 0.38 mm^−1^
*T* = 100.0 (1) K
0.55 × 0.37 × 0.17 mm

#### Data collection

Bruker SMART APEXII CCD area-detector diffractometer
Absorption correction: multi-scan (*SADABS*; Bruker, 2005 ▶) *T*_min_ = 0.821, *T*_max_ = 0.938
16377 measured reflections
3160 independent reflections
2760 reflections with *I* > 2σ(*I*)
*R*_int_ = 0.029

#### Refinement

*R*[*F* ^2^ > 2σ(*F* ^2^)] = 0.032
*wR*(*F* ^2^) = 0.087
*S* = 1.05
3160 reflections
104 parameters
H atoms treated by a mixture of independent and constrained refinement
Δρ_max_ = 0.55 e Å^−3^
Δρ_min_ = −0.23 e Å^−3^

### 

Data collection: *APEX2* (Bruker, 2005 ▶); cell refinement: *APEX2*; data reduction: *SAINT* (Bruker, 2005 ▶); program(s) used to solve structure: *SHELXTL* (Sheldrick, 2008 ▶); program(s) used to refine structure: *SHELXTL*; molecular graphics: *SHELXTL*; software used to prepare material for publication: *SHELXTL* and *PLATON* (Spek, 2003 ▶).

## Supplementary Material

Crystal structure: contains datablocks global, I. DOI: 10.1107/S1600536809003286/tk2360sup1.cif

Structure factors: contains datablocks I. DOI: 10.1107/S1600536809003286/tk2360Isup2.hkl

Additional supplementary materials:  crystallographic information; 3D view; checkCIF report

## Figures and Tables

**Table 1:** Hydrogen-bond geometry (Å, °)

*D*—H⋯*A*	*D*—H	H⋯*A*	*D*⋯*A*	*D*—H⋯*A*
N3—H1N3⋯N1^i^	0.84 (2)	2.24 (2)	3.070 (1)	172 (1)
N4—H1N4⋯N2^ii^	0.82 (2)	2.42 (2)	3.208 (1)	161 (1)
N4—H2N4⋯N2^iii^	0.89 (1)	2.30 (2)	3.137 (1)	157 (1)

Symmetry codes: (i) 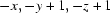; (ii) 
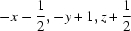
; (iii) 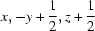.

**Fig. 1.**
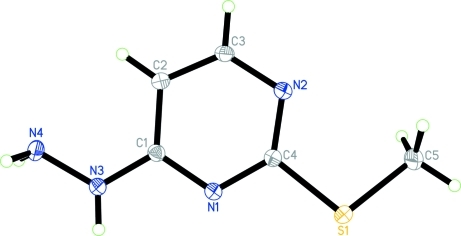
The molecular structure of (I), showing 50% probability displacement ellipsoids and the atom-numbering scheme.

**Fig. 2.**
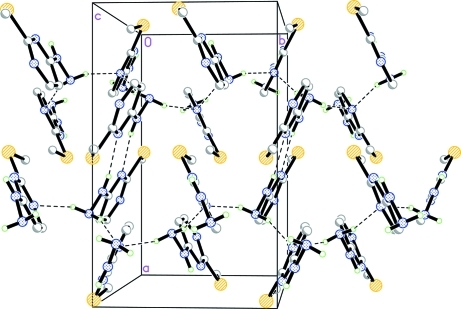
A view of the crystal packing in (I), viewed down the c axis, showing wave-like chains along the b axis. H atoms involved in hydrogen bonds are shown as dotted lines. Other H atoms have been omitted for clarity.

## supplementary crystallographic information

### Comment

Pyrimidines and their derivatives possess biological and pharmacological activities such as antibacterial, antimicrobial, anti-inflammatory, analgesic, anticonvulsant and anti-aggressive activities (Ghorab *et al.*, 2004; Anderson *et al.*, 1990). This prompted us to synthesize compounds bearing the pyrimidine moiety. Hydrazine derivatives are interesting building blocks of heterocyclic compounds containing N—N bonds (Geza *et al.*, 1981; Gante, 1989). Some hydrazine derivatives such as phthalazin-1-yl-hydrazine are widely used as general antihypertensive and vasodilator agents, and are considered as a first-line drug in the management of pregnancy-induced hypertension (Powers *et al.*, 1998; Vidrio *et al.*, 2003). In addition, these compounds are known to decompose easily in the presence of radicals into hydrazine derivatives which are commonly used as rocket fuels. The structure of the title compound, (I), was determined in this context. The molecule of (I), Fig. 1, is essentially planar, with the maximum deviation from the least-squares plane being 0.297 (1) Å for the C5 atom.

The primary interactions in the crystal structure are of the type N—H···N, Table 1 and Fig. 2. Here, molecules form wave-like supramolecular chains along the *b* axis with successive molecules connected on either side via *R*_2_^2^(8) motifs (Bernstein *et al.*, 1995) to form a 2-D array.

### Experimental

4-Chloro-2-(methylsulfanyl)pyrimidine (0.01 mol) was dissolved in methanol and 99% hydrazine hydrate (0.015 mol) was added dropwise with external cooling. The mixture was stirred at room temperature for 5 h. The precipitate was filtered, dried and recrystallized from ethyl acetate. Crystals suitable for X-ray studies are obtained from ethyl acetate by slow evaporation. Yield 65%, m.p. 413 K.

### Refinement

All H atoms were positioned geometrically and refined with a riding model approximation with C—H = 0.93–0.96 Å, and with U_iso_(H) = 1.2–1.5U_eq_(C). The rotating model group was employed for the methyl group. In the case of N3 and N4 atoms, the H atoms were located from a difference Fourier map and refined isotropically, see Table 1 for bond distances.

### Crystal data

**Table:**

C_5_H_8_N_4_S	*F*(000) = 656
*M**_r_* = 156.21	*D*_x_ = 1.446 Mg m^−^^3^
Orthorhombic, *P**b**c**a*	Mo *K*α radiation, λ = 0.71073 Å
Hall symbol: -P 2ac 2ab	Cell parameters from 6385 reflections
*a* = 12.7906 (2) Å	θ = 3.2–38.6°
*b* = 7.7731 (1) Å	µ = 0.38 mm^−^^1^
*c* = 14.4354 (3) Å	*T* = 100 K
*V* = 1435.21 (4) Å^3^	Block, colourless
*Z* = 8	0.55 × 0.37 × 0.17 mm

### Data collection

**Table:**

Bruker SMART APEXII CCD area-detector diffractometer	3160 independent reflections
Radiation source: fine-focus sealed tube	2760 reflections with *I* > 2σ(*I*)
graphite	*R*_int_ = 0.029
φ and ω scans	θ_max_ = 35.0°, θ_min_ = 2.8°
Absorption correction: multi-scan (*SADABS*; Bruker, 2005)	*h* = −20→19
*T*_min_ = 0.821, *T*_max_ = 0.938	*k* = −12→10
16377 measured reflections	*l* = −15→23

### Refinement

**Table:**

Refinement on *F*^2^	Primary atom site location: structure-invariant direct methods
Least-squares matrix: full	Secondary atom site location: difference Fourier map
*R*[*F*^2^ > 2σ(*F*^2^)] = 0.032	Hydrogen site location: inferred from neighbouring sites
*wR*(*F*^2^) = 0.087	H atoms treated by a mixture of independent and constrained refinement
*S* = 1.05	*w* = 1/[σ^2^(*F*_o_^2^) + (0.0429*P*)^2^ + 0.4069*P*] where *P* = (*F*_o_^2^ + 2*F*_c_^2^)/3
3160 reflections	(Δ/σ)_max_ < 0.001
104 parameters	Δρ_max_ = 0.55 e Å^−^^3^
0 restraints	Δρ_min_ = −0.23 e Å^−^^3^

### Special details

**Table:**

Experimental. The data was collected with the Oxford Cryosystem Cobra low-temperature attachment
Geometry. All e.s.d.'s (except the e.s.d. in the dihedral angle between two l.s. planes) are estimated using the full covariance matrix. The cell e.s.d.'s are taken into account individually in the estimation of e.s.d.'s in distances, angles and torsion angles; correlations between e.s.d.'s in cell parameters are only used when they are defined by crystal symmetry. An approximate (isotropic) treatment of cell e.s.d.'s is used for estimating e.s.d.'s involving l.s. planes.
Refinement. Refinement of *F*^2^ against ALL reflections. The weighted *R*-factor *wR* and goodness of fit *S* are based on *F*^2^, conventional *R*-factors *R* are based on *F*, with *F* set to zero for negative *F*^2^. The threshold expression of *F*^2^ > σ(*F*^2^) is used only for calculating *R*-factors(gt) *etc*. and is not relevant to the choice of reflections for refinement. *R*-factors based on *F*^2^ are statistically about twice as large as those based on *F*, and *R*- factors based on ALL data will be even larger.

### Fractional atomic coordinates and isotropic or equivalent isotropic displacement parameters (Å^2^)

**Table:**

	*x*	*y*	*z*	*U*_iso_*/*U*_eq_	
S1	0.009609 (17)	0.58095 (3)	0.239703 (15)	0.01516 (6)	
N1	−0.07742 (5)	0.47877 (10)	0.39095 (5)	0.01315 (13)	
N2	−0.17805 (6)	0.42798 (10)	0.25325 (5)	0.01321 (13)	
N3	−0.13619 (6)	0.40510 (11)	0.53498 (5)	0.01709 (15)	
N4	−0.21443 (6)	0.34229 (11)	0.59510 (5)	0.01670 (14)	
C1	−0.15419 (6)	0.40931 (10)	0.44356 (6)	0.01236 (14)	
C2	−0.24631 (6)	0.34361 (11)	0.40217 (6)	0.01379 (14)	
H2A	−0.2994	0.2943	0.4373	0.017*	
C3	−0.25315 (6)	0.35641 (11)	0.30784 (6)	0.01370 (14)	
H3A	−0.3128	0.3135	0.2792	0.016*	
C4	−0.09458 (6)	0.48345 (10)	0.29943 (5)	0.01199 (13)	
C5	−0.01837 (8)	0.53439 (15)	0.12034 (7)	0.02202 (19)	
H5A	0.0365	0.5797	0.0820	0.033*	
H5B	−0.0836	0.5865	0.1033	0.033*	
H5C	−0.0229	0.4121	0.1119	0.033*	
H1N3	−0.0801 (12)	0.4477 (18)	0.5550 (10)	0.027 (4)*	
H1N4	−0.2343 (11)	0.420 (2)	0.6293 (10)	0.025 (4)*	
H2N4	−0.1890 (10)	0.2573 (19)	0.6296 (10)	0.024 (3)*	

### Atomic displacement parameters (Å^2^)

**Table:**

	*U*^11^	*U*^22^	*U*^33^	*U*^12^	*U*^13^	*U*^23^
S1	0.01382 (10)	0.01761 (11)	0.01405 (10)	−0.00356 (7)	0.00068 (6)	0.00078 (7)
N1	0.0126 (3)	0.0153 (3)	0.0115 (3)	−0.0011 (2)	−0.0007 (2)	−0.0003 (2)
N2	0.0126 (3)	0.0147 (3)	0.0123 (3)	−0.0004 (2)	−0.0012 (2)	−0.0003 (2)
N3	0.0135 (3)	0.0266 (4)	0.0111 (3)	−0.0045 (3)	−0.0009 (2)	0.0012 (3)
N4	0.0149 (3)	0.0223 (4)	0.0129 (3)	−0.0009 (3)	0.0026 (2)	0.0012 (3)
C1	0.0119 (3)	0.0134 (3)	0.0117 (3)	0.0008 (2)	−0.0004 (2)	−0.0003 (3)
C2	0.0119 (3)	0.0155 (3)	0.0140 (3)	−0.0016 (3)	−0.0004 (2)	−0.0001 (3)
C3	0.0118 (3)	0.0150 (3)	0.0143 (3)	−0.0007 (3)	−0.0017 (2)	−0.0008 (3)
C4	0.0119 (3)	0.0117 (3)	0.0123 (3)	0.0006 (2)	0.0001 (2)	−0.0003 (2)
C5	0.0187 (4)	0.0334 (5)	0.0139 (4)	−0.0036 (4)	0.0017 (3)	0.0008 (3)

### Geometric parameters (Å, °)

**Table:**

S1—C4	1.7589 (8)	N4—H1N4	0.822 (15)
S1—C5	1.7967 (10)	N4—H2N4	0.889 (15)
N1—C4	1.3397 (10)	C1—C2	1.4164 (11)
N1—C1	1.3537 (11)	C2—C3	1.3681 (12)
N2—C4	1.3305 (11)	C2—H2A	0.9300
N2—C3	1.3613 (11)	C3—H3A	0.9300
N3—C1	1.3399 (11)	C5—H5A	0.9600
N3—N4	1.4118 (11)	C5—H5B	0.9600
N3—H1N3	0.841 (15)	C5—H5C	0.9600
			
C4—S1—C5	103.44 (4)	C1—C2—H2A	121.7
C4—N1—C1	116.44 (7)	N2—C3—C2	124.11 (8)
C4—N2—C3	114.11 (7)	N2—C3—H3A	117.9
C1—N3—N4	119.48 (7)	C2—C3—H3A	117.9
C1—N3—H1N3	118.4 (10)	N2—C4—N1	128.08 (8)
N4—N3—H1N3	121.9 (10)	N2—C4—S1	120.13 (6)
N3—N4—H1N4	109.5 (10)	N1—C4—S1	111.77 (6)
N3—N4—H2N4	110.0 (9)	S1—C5—H5A	109.5
H1N4—N4—H2N4	109.0 (13)	S1—C5—H5B	109.5
N3—C1—N1	115.96 (7)	H5A—C5—H5B	109.5
N3—C1—C2	123.34 (8)	S1—C5—H5C	109.5
N1—C1—C2	120.70 (7)	H5A—C5—H5C	109.5
C3—C2—C1	116.54 (8)	H5B—C5—H5C	109.5
C3—C2—H2A	121.7		
			
N4—N3—C1—N1	176.97 (8)	C1—C2—C3—N2	−0.32 (13)
N4—N3—C1—C2	−4.05 (13)	C3—N2—C4—N1	−0.91 (12)
C4—N1—C1—N3	179.94 (8)	C3—N2—C4—S1	−179.53 (6)
C4—N1—C1—C2	0.92 (12)	C1—N1—C4—N2	−0.07 (13)
N3—C1—C2—C3	−179.68 (8)	C1—N1—C4—S1	178.65 (6)
N1—C1—C2—C3	−0.74 (12)	C5—S1—C4—N2	−12.16 (8)
C4—N2—C3—C2	1.08 (12)	C5—S1—C4—N1	169.01 (6)

### Hydrogen-bond geometry (Å, °)

**Table:**

*D*—H···*A*	*D*—H	H···*A*	*D*···*A*	*D*—H···*A*
N3—H1N3···N1^i^	0.84 (2)	2.24 (2)	3.070 (1)	172 (1)
N4—H1N4···N2^ii^	0.82 (2)	2.42 (2)	3.208 (1)	161 (1)
N4—H2N4···N2^iii^	0.89 (1)	2.30 (2)	3.137 (1)	157 (1)

Symmetry codes: (i) −*x*, −*y*+1, −*z*+1; (ii) −*x*−1/2, −*y*+1, *z*+1/2; (iii) *x*, −*y*+1/2, *z*+1/2.
